# Intestinal permeability biomarkers in patients with schizophrenia: Additional support for the impact of lifestyle habits

**DOI:** 10.1192/j.eurpsy.2024.1765

**Published:** 2024-12-16

**Authors:** Leticia González-Blanco, Francesco Dal Santo, Maria Paz García-Portilla, Miqueu Alfonso, Carla Hernández, Mónica Sánchez-Autet, Gerard Anmella, Silvia Amoretti, Gemma Safont, David Martín-Hernández, Stefanie Malan-Müller, Miquel Bernardo, Belén Arranz

**Affiliations:** 1 Área de Psiquiatría, Universidad de Oviedo, Oviedo, Spain; 2 Servicio de Salud del Principado de Asturias (SESPA), Oviedo, Spain; 3 Instituto de Investigación Sanitaria del Principado de Asturias (ISPA), Oviedo, Spain; 4 Instituto de Neurociencias del Principado de Asturias (INEUROPA), Oviedo, Spain; 5 Biomedical Research Networking Center for Mental Health Network (CIBERSAM), Institute of Health Carlos III, Madrid, Spain; 6 Parc Sanitari Sant Joan de Deu, Barcelona, Spain; 7 Department of Psychiatry and Psychology, Institute of Neuroscience, Hospital Clínic de Barcelona, Barcelona, Spain; 8 Bipolar and Depressive Disorders Unit, Digital Innovation Group, Institut d’Investigacions Biomèdiques August Pi i Sunyer (IDIBAPS), Barcelona, Spain; 9 Department of Medicine, School of Medicine and Health Sciences, Institute of Neurosciences (UBNeuro), University of Barcelona (UB), Barcelona, Spain; 10 Barcelona Clinic Schizophrenia Unit, Hospital Clinic, Departament de Medicina, Institut de Neurociències (UBNeuro), Universitat de Barcelona (UB), Barcelona, Spain; 11 Institut d’Investigacions Biomèdiques August Pi I Sunyer (IDIBAPS), ISCIII, Barcelona, Spain; 12 Department of Psychiatry, Hospital Universitari Vall d’Hebron, Barcelona, Spain; 13 Group of Psychiatry, Mental Health and Addictions, Psychiatric Genetics Unit, Vall d’Hebron Research Institute (VHIR), Barcelona, Spain; 14 Universitat Autònoma de Barcelona, Barcelona, Spain; 15 Department of Psychiatry, Hospital Universitari Mútua Terrassa, ISIC Medical Center, Barcelona, Spain; 16 University of Barcelona, Barcelona, Spain; 17 Department of Pharmacology and Toxicology, Faculty of Medicine, University Complutense Madrid (UCM), Madrid, Spain; 18 Hospital 12 de Octubre Research Institute (Imas12), Madrid, Spain; 19 Neurochemistry Research Institute UCM, Madrid, Spain

**Keywords:** cognition, diet, intestinal permeability, psychopathology, schizophrenia

## Abstract

**Background:**

Emerging evidence suggests a potential association between “leaky gut syndrome” and low-grade systemic inflammation in individuals with psychiatric disorders, such as schizophrenia. Gut dysbiosis could increase intestinal permeability, allowing the passage of toxins and bacteria into the systemic circulation, subsequently triggering immune-reactive responses. This study delves into understanding the relationship between plasma markers of intestinal permeability and symptom severity in schizophrenia. Furthermore, the influence of lifestyle habits on these intestinal permeability markers was determined.

**Methods:**

Biomarkers of intestinal permeability, namely lipopolysaccharide-binding protein (LBP), lipopolysaccharides (LPS), and intestinal fatty acid binding protein (I-FABP), were analyzed in 242 adult schizophrenia patients enrolled in an observational, cross-sectional, multicenter study from four centers in Spain (PI17/00246). Sociodemographic and clinical data were collected, including psychoactive drug use, lifestyle habits, the Positive and Negative Syndrome Scale to evaluate schizophrenia symptom severity, and the Screen for Cognitive Impairment in Psychiatry to assess cognitive performance.

**Results:**

Results revealed elevated levels of LBP and LPS in a significant proportion of patients with schizophrenia (62% and 25.6%, respectively). However, no statistically significant correlation was observed between these biomarkers and the overall clinical severity of psychotic symptoms or cognitive performance, once confounding variables were controlled for. Interestingly, adherence to a Mediterranean diet was negatively correlated with I-FABP levels (*beta* = −0.186, *t* = −2.325, *p* = 0.021), suggesting a potential positive influence on intestinal barrier function.

**Conclusions:**

These findings underscore the importance of addressing dietary habits and promoting a healthy lifestyle in individuals with schizophrenia, with potential implications for both physical and psychopathological aspects of the disorder.

## Introduction

Schizophrenia is a complex, heterogeneous syndrome that impacts behavior and cognition and is linked to a high prevalence of comorbid systemic conditions. The disorder results from genetic and environmental factors and their interplay with the developing brain’s environment [1], with some of these risk factors linked to immunological processes. Several studies have observed increased inflammatory markers such as interleukin (IL)-6 and tumor necrosis factor alpha, along with reduced IL-10 in patients with schizophrenia [2]. Accordingly, a low-grade inflammatory state, characterized by slightly increased systemic levels of C-reactive protein, has been hypothesized in patients with schizophrenia and other psychoses [3].

In recent years, there has been emerging evidence that “leaky gut syndrome” could be a potential contributor to low-grade systemic inflammation observed in patients with different psychiatric disorders. It may also contribute to metabolic complications, including obesity and type 2 diabetes [4–7]. The bidirectional communication pathway known as the gut–brain axis has been implicated in various neuropsychiatric conditions, with its effects mediated through gut dysbiosis or an imbalance of the gut microbiome [8]. Gut dysbiosis could increase intestinal permeability, leading to the development of “leaky gut syndrome,” and thereby allowing the passage of toxins and bacteria into the systemic circulation, subsequently triggering immune-reactive responses.

In particular, a leaky intestinal barrier allows the translocation of lipopolysaccharides (LPS), endotoxins found on the membrane of gram-negative bacteria, from the gut into the peripheral circulation [9]. Consequently, LPS stimulates various immune cells, acting through lipopolysaccharide-binding protein (LBP), and leading to increased secretion of pro-inflammatory cytokines and systemic low-grade inflammation [10]. LBP is an acute-phase protein, mainly secreted in the liver with a longer half-life, that binds to bacterial LPS and is considered a useful biomarker for intestinal permeability [11]. However, the levels of LPS or LBP are not the only markers of intestinal permeability, zonulin and intestinal fatty acid binding protein (I-FABP) are indirect biomarkers of gut barrier dysfunction [12]. Higher levels of these parameters have been found in patients with schizophrenia and affective disorders [13–15]. Nevertheless, research examining the relationship between intestinal permeability and the psychopathology of schizophrenia across different symptomatic domains is currently limited. Furthermore, dietary and toxic habits have been related to intestinal barrier integrity, but this has been poorly studied in patients with mental disorders [16, 17].

In this context, the objectives of the study were (a) to measure intestinal permeability in a cohort of patients with schizophrenia through plasma markers related to bacterial translocation – LBP, LPS, and I-FABP; (b) to investigate its association with the clinical severity of schizophrenia across the main symptomatic domains including positive symptoms, negative symptoms, and cognitive performance; and (c) to analyze the impact of lifestyle habits on these biomarkers.

## Methods

The present study finally included 242 adults with a confirmed diagnosis of schizophrenia according to DSM-5 (Diagnostic and Statistical Manual of Mental Disorders) criteria [18]. The sample came from an observational, cross-sectional, multicenter study at four centers in Spain (PI17/00246), with a larger sample of adult patients with DSM-5 schizophrenia spectrum disorder at any stage of the disease. Its objectives and protocol have been previously described [19]. All patients provided written informed consent to participate. The study protocol was approved by the local ethics committees at the participating centers (127/2015).

The inclusion criteria consisted of (1) adults over 18 years old, (2) Spanish verbal fluency, and (3) providing signed informed consent. Exclusion criteria included (1) past head trauma resulting in loss of consciousness, (2) organic diseases with mental repercussions, and (3) acute inflammatory events (e.g., fever >38°C or infection in the 2 weeks preceding the interview or vaccines in the previous 4 weeks).

### Clinical and sociodemographic assessment

Baseline data were collected, including sociodemographic variables, years of illness duration, toxic habits, psychoactive drug use, and anthropometric measurements (weight, height, and body mass index [BMI]).

The Spanish version of the Positive and Negative Syndrome Scale (PANSS) was used to assess psychopathology, with higher scores indicating greater severity [20]. To assess cognitive impairment, the Screen for Cognitive Impairment in Psychiatry was used, evaluating immediate and delayed verbal learning, working memory, verbal fluency, and processing speed [21]. In the five neurocognitive domains, higher scores correspond to better performance. The cognitive evaluation was only available in 163 patients. The functioning level was assessed by the Global Assessment of Functioning (GAF), with higher scores indicating better functioning [22].

Finally, lifestyle habits were assessed with the Mediterranean Diet Adherence Screener (MEDAS) [23, 24], a validated questionnaire of Mediterranean diet adherence consisting of 14 items (intake and amount of extra-virgin olive oil, frequency of fruit, vegetables, nuts, legumes, red meat, poultry, fish, animal fat, sweetened beverages, sweets, and fried food), used in the Prevención con Dieta Mediterránea study [25]. MEDAS score was calculated by assigning a score of 1 and 0 for each item. Also, lifestyle habits were assessed with the Short Scale of Physical Activity (IPAQ) [26], with IPAQ “activity” assessing specific types of activities (walking, moderate-intensity activities, and vigorous-intensity activities) with results expressed as MET-min per week, and IPAQ “sitting” (sedentary) assessing time spent sitting (minutes per week). Additionally, toxic habits were collected in the *ad hoc* interview, self-reported by the patient.

### Laboratory assessment

After a confirmed overnight fast, two 10 mL tubes of peripheral blood were obtained by venipuncture and processed to obtain serum, which was used to quantify LBP, LPS, and I-FABP levels, following the manufacturer’s protocol for commercially available kits (RayBiotech Human LBP ELISA Kit ELH-LBP, Hycult Biotech LAL Chromogenic Endpoint Assay #HIT302, Cusabio iFABP ELISA Kit CSB-E08024h).

### Statistical analyses

First, descriptive analyses of the sociodemographic, clinical, and biological characteristics of the sample were performed. The normality of continuous variables was tested using the Kolmogorov–Smirnov test.

Second, Mann–Whitney *U* tests and Spearman correlations were used to explore associations between intestinal permeability markers and clinical scores. Partial correlations were performed to explore previous significant associations, adjusting for covariates. Moreover, linear multiple regression analyses were performed to explore the impact of lifestyle variables on bacterial translocation markers when univariate analyses were statistically significant.

All statistical analyses were performed using IBM Statistical Package for the Social Sciences (SPSS) v.27. Two-tailed *p-*values <0.05 were considered statistically significant.

## Results

### Sociodemographic and clinical characteristics

Of the total sample (*N* = 242), 63.6% were male and the mean age was 44.09 (range: 18–76) years. Patients had a mean duration of illness of 16.04 years with a standard deviation (SD) of 10.78 years. Substance use was reported by 50.7% of the sample, primarily tobacco (44.8%), alcohol (21.3%), and cannabis (7.9%). All but one patient received at least one antipsychotic, with 36.7% receiving two or more antipsychotics, and 33.6% receiving a long-acting injectable. Antidepressants were used by 32.8% of patients, and 6.6% received a mood stabilizer. The sociodemographic, clinical, and psychometric characteristics of the sample are detailed in Table 1.

**Table 1.** Sociodemographic, clinical, anthropometric, and biological data of the sample. Data are expressed as mean (SD) or *n* (%)

	Total sample (*n* = 242)
Age (years)	44.09 (12.63)
Sex, male	154 (63.6%)
Illness duration (years)	16.04 (10.78)
Smoking (yes)	104 (44.8%)
Cigarettes per day (if smoking)	17.64 (10.10)
Alcohol (yes)	46 (21.3%)
SDUs/week (if used)	5.15 (6.50)
BMI (kg/m^2^)	28.42 (5.83)
CRP (mg/dL)	0.54 (0.84)
LBP (μg/mL)	17.93 (7.53)
LPS (EU/mL)	0.76 (2.43)
I-FABP (ng/mL)	0.23 (0.44)
*Clinical variables*	
PANSS Positive	12.68 (5.27)
PANSS Negative	20.81 (7.54)
PANSS GP	30.28 (9.17)
PANSS Total Score	63.80 (18.65)
SCIP Total Score^a^	57.40 (16.93)
Immediate verbal learning^a^	17.15 (4.93)
Delayed verbal learning^a^	15.00 (5.85)
Working memory^a^	13.42 (5.22)
Verbal fluency^a^	4.18 (2.52)
Processing speed^a^	7.66 (3.61)
GAF	55.44 (13.93)
MEDAS score	7.68 (2.22)
IPAQ activity (MET-min/week) (*n* = 194)	1,905.35 (2,403.19)
IPAQ sitting (minutes/week) (*n* = 182)	2,552.64 (1,493.87)

Abbreviations: BMI, body mass index; CRP, C-reactive protein; GAF, Global Assessment of Functioning; GP, general psychopathology; I-FABP, intestinal fatty acid binding protein; IPAQ, International Physical Activity Questionnaire; LBP, lipopolysaccharide-binding protein; LPS, lipopolysaccharides; MEDAS, Mediterranean Diet Adherence Screener; PANSS, Positive and Negative Syndrome Scale; SDUs, standard drink units.

a *n* = 163.

### Intestinal permeability markers

Mean plasma concentrations of LBP (μg/mL), LPS (EU/mL), and I-FABP (ng/mL) are shown in Table 1. The three variables exhibited non-normal distributions (*p* < 0.05). Based on reference values, elevated intestinal permeability, defined as levels of LBP >15 μg/mL [27], was present in 62% of the sample. Also, 25.6% had LPS concentrations above 0.5 EU/mL [28], and 18.9% showed increased levels of both LBP and LPS simultaneously. However, only two patients had levels of I-FABP >2 ng/mL based on reference values [29].

No statistically significant differences in gut permeability markers were noted between the sexes. We observed a positive correlation between age and LBP (*r* = 0.162, *p* = 0.012) and between longer duration of illness and LBP (*r* = 0.141, *p* = 0.041) and I-FABP (*r* = 0.167, *p* = 0.016). Also, BMI was positively correlated with LBP (*r* = 0.182, *p* = 0.004) and LPS (*r* = 0.298, *p* < 0.001).

No differences were detected based on psychoactive drugs (*p* > 0.05), except that higher LPS values were evident in patients using antidepressants (1.16 (3.59) vs. 0.57 (1.59); *U* = 4,971.5, *p* = 0.016).

#### Intestinal permeability markers and psychopathological domains

As presented in Table 2, I-FABP levels were weakly, but significantly correlated with symptom severity, as measured by the PANSS total score. None of the permeability markers were significantly correlated with positive symptoms, while both LPS and I-FABP plasma levels were positively correlated with negative symptoms. Furthermore, a significant positive correlation was noted between I-FABP levels and the PANSS General Psychopathology score. Next, we performed partial correlations adjusting for age and BMI, finding no statistically significant associations between any of the biomarkers and clinical scores (*p* > 0.05).

**Table 2.** Intestinal permeability markers and clinical domains. Data are expressed as the correlation coefficient, *r*, and significance (*p*-value)

	LBP (μg/mL)	LPS (EU/mL)	I-FABP (ng/mL)
PANSS Positive	−0.029 (0.649)	0.061 (0.345)	0.092 (0.155)
PANSS Negative	0.068 (0.293)	0.145 (0.026)	0.179 (0.006)
PANSS GP	0.054 (0.402)	0.037 (0.571)	0.149 (0.021)
PANSS Total Score	0.039 (0.542)	0.084 (0.197)	0.185 (0.004)
SCIP Total Score	−0.102 (0.197)	−0.110 (0.165)	−0.096 (0.229)
Immediate verbal learning^a^	−0.067 (0.395)	−0.061 (0.445)	−0.152 (0.054)
Delayed verbal learning^a^	−0.198 (0.011)	−0.131 (0.099)	−0.147 (0.063)
Working memory^a^	0.043 (0.589)	0.026 (0.745)	0.113 (0.153)
Verbal fluency^a^	−0.120 (0.126)	−0.112 (0.156)	−0.082 (0.304)
Processing speed^a^	−0.100 (0.204)	−0.110 (0.166)	−0.083 (0.295)
GAF	0.064 (0.356)	−0.112 (0.107)	0.03 (0.971)

Abbreviations: GAF, Global Assessment of Functioning; GP, general psychopathology; I-FABP, intestinal fatty acid binding protein; IPAQ, International Physical Activity Questionnaire; LBP, lipopolysaccharide-binding protein; LPS, lipopolysaccharides; MEDAS, Mediterranean Diet Adherence Screener; PANSS, Positive and Negative Syndrome Scale.

a *n* = 163.

#### Intestinal permeability markers and cognitive performance

For neurocognitive functioning, we observed a negative correlation between LBP levels and delayed verbal learning (Table 3). However, following partial correlations controlling for the effect of age and BMI, this correlation was no longer statistically significant.

**Table 3.** Spearman correlations between intestinal permeability markers and lifestyle habits (*r* and *p*-values)

	LBP (μg/mL)	LPS (EU/mL)	I-FABP (ng/mL)
MEDAS score	−0.010 (0.884)	−0.081 (0.237)	−0.149 (0.030)
IPAQ activity (MET-min/week)	−0.028 (0.701)	−0.091 (0.212)	−0.101 (0.164)
IPAQ sitting (minutes/week)	0.049 (0.508)	−0.142 (0.059)	0.178 (0.018)
Smoking (cigarettes per day)	0.069 (0.293)	0.285 (<0.001)	0.178 (0.007)
Alcohol consumption (SDUs/week)	−0.073 (0.287)	0.020 (0.771)	0.079 (0.255)

Abbreviations: I-FABP, intestinal fatty acid binding protein; IPAQ, International Physical Activity Questionnaire; LBP, lipopolysaccharide-binding protein; LPS, lipopolysaccharides; MEDAS, Mediterranean Diet Adherence Screener; SDUs, standard drink units.

### Impact of lifestyle habits on intestinal permeability markers

Spearman correlations between intestinal permeability markers and lifestyle variables are reported in Table 3. Levels of I-FABP were significantly correlated with MEDAS total score, with increased plasma levels associated with lower adherence to a Mediterranean diet (*r* = −0.149, *p* = 0.030). IPAQ activity did not correlate with any of the intestinal markers, but a higher score on IPAQ sitting (sedentary lifestyle) was correlated with increased levels of I-FABP (*r* = 0.178, *p* = 0.018). Regarding toxic habits, the number of cigarettes smoked per day was positively correlated with LPS (*r* = 0.285, *p* < 0.001) and I-FABP (*r* = 0.178, *p* = 0.007). However, alcohol consumption did not have an impact on intestinal permeability.

Multiple linear regression analyses were performed for both markers, LPS and I-FABP, considering all variables, including psychopathological sub-scores, associated with each parameter in the univariate analyses. A model was obtained for I-FABP (*F* = 5.407, *p* = 0.021, *R^2^* = 0.035), where only the MEDAS score entered as a predictive variable (*beta* = −0.186, *t* = −2.325, *p* = 0.021). Furthermore, none of the variables entered in the model for LPS. Since no lifestyle variable was found to be associated with LBP, no model was performed for this marker.

## Discussion

The objective of this article was to evaluate the intestinal integrity of patients with schizophrenia, by analyzing plasma biomarkers of intestinal permeability or bacterial translocation: LBP, LPS, and I-FABP. Additionally, we aimed to determine if increased intestinal permeability would be related to greater clinical severity of psychotic symptoms and worse cognitive function and to explore the impact of lifestyle habits, such as diet, physical activity, and substance use, on these biomarkers.

Our main results suggest that a large percentage of schizophrenia patients have increased intestinal permeability, as indicated by elevated concentrations of LBP.

Previous studies have reported increased bacterial translocation and barrier dysfunction in patients with schizophrenia. In the study by Gokulakrishnan et al. [14], LBP levels were significantly higher in schizophrenia patients compared with healthy controls. Furthermore, in a large cohort of patients with severe mental illness, including 389 patients with schizophrenia, LBP and I-FAPB were higher compared with healthy controls [15]. However, the highest levels of both peripheral biomarkers were found in affective disorders.

Another important result is that no direct relationship was identified between these markers and the clinical severity of psychotic symptoms overall, in either positive or negative symptoms or cognitive performance, once potential confounding factors were controlled for. Nor was any correlation observed with the level of functioning, as evaluated with the GAF. Limited data are available that report on this association; however, studies mainly report negative results [14, 30]. Our results contrast with a recent study where a slight correlation was detected between LBP levels and PANSS total score when considering CRP and BMI as confounding factors [15]. In the study by Dal Santo et al. [31] using network techniques in a similar cohort of patients, LBP was positively associated with CRP and BMI, but only indirectly connected to psychopathology.

The relationship between intestinal permeability and cognitive function in schizophrenia is under-investigated [32]. In keeping with our results, LBP (nor sCD14) had no direct correlation with Brief Assessment of Cognition in Schizophrenia (BACS) scores in patients with schizophrenia spectrum disorders in a recent study [33]. However, in the healthy group, both biomarkers were indirectly associated with decreased cognition, with intracranial volume as a mediator. These authors concluded that increased bacterial translocation may negatively affect brain volume, which consequently impacts cognition. On the other hand, Ishida et al. [30] reported that patients with schizophrenia had higher rates of gut permeability, as measured with the lactulose-to-mannitol ratio test, and that this index was negatively correlated with the BACS total score.

Another interesting finding in the present study was the inverse relationship between I-FABP plasma concentrations and higher scores on the MEDAS, suggesting that a healthier diet may have a beneficial effect on the intestinal barrier, as has been widely documented [16, 34]. It is worth noting that, in accordance with the predefined threshold [29], only two patients had elevated levels of this parameter. Nevertheless, I-FABP is a sensitive indicator of enterocyte damage, and it is released into the systemic circulation when there is an injury to intestinal epithelial cells [35]. Although the usefulness of this marker has not been established in psychiatric samples, a recent meta-analysis by Arnone [36] combined results from several studies concluding that increased peripheral levels of I-FABP might contribute to gastrointestinal permeability in mood disorders. Furthermore, Ohlsson et al. [37] found altered levels of I-FABP in patients with recent suicide attempts.

Both LPS and LBP levels were positively correlated with BMI in our sample. It is worth noting that obesity has previously been associated with altered gut microbiota and low-grade inflammation [38]. Indeed, there is evidence of an association of gut dysbiosis in both schizophrenia and obesity, indicating possible common shared pathophysiological mechanisms related to immune inflammation [39]. Moreover, other metabolic complications, such as type 2 diabetes, have been related to increased intestinal permeability in clinical samples [40]. We consider that no direct relationship between intestinal permeability and psychopathology was observed, as BMI might be an intermediary, with an impact on cognitive and negative symptoms as previously reported [41, 42].

Some limitations of the present study should be mentioned. The parameters analyzed provide an indirect measure of intestinal permeability. Specifically, LBP concentrations reflect a hepatic response intended to neutralize LPS translocation across the gastrointestinal barrier [12]. Other factors can also affect this parameter; therefore, we controlled for several variables such as diet, physical exercise, smoking and other toxic habits, and BMI. In addition, there is no healthy control group and the cross-sectional design of the study prevents inferring causality of the results.

It bears mentioning that, although diet and physical exercise were self-reported, the psychometric evaluation was carried out by trained psychologists or psychiatrists, covering all the symptomatic domains of schizophrenia (including cognition). Another strength is that this is a naturalistic, multicenter study with a substantial and heterogeneous patient cohort under treatment. As differences between bipolar disorder and schizophrenia patients have been reported [15, 43], only patients with schizophrenia were included in this analysis to avoid bias.

## Conclusions

We report evidence of increased levels of intestinal permeability biomarkers, specifically LBP and LPS, in patients with schizophrenia. However, we did not find an association between these biomarkers and clinical severity in different domains. A Mediterranean diet could have a positive influence on gut barrier function. It is important to address dietary habits in these individuals, prioritizing a healthy lifestyle. This could have an impact on altered gut permeability with implications for physical comorbidities and probably an indirect influence on psychopathological aspects of the disorder.
